# Use of artificial intelligence to develop predictive algorithms of cough and PCR-confirmed COVID-19 infections based on inputs from clinical-grade wearable sensors

**DOI:** 10.1038/s41598-024-57830-4

**Published:** 2024-04-05

**Authors:** Jessica R. Walter, Jong Yoon Lee, Lian Yu, Brandon Kim, Knute Martell, Anita Opdycke, Jenny Scheffel, Ingrid Felsl, Soham Patel, Stephanie Rangel, Alexa Serao, Claire Edel, Ankit Bharat, Shuai Xu

**Affiliations:** 1 Department of Obstetrics and Gynecology, Northwestern University, Chicago, IL USA; 2 Sibel Health, Chicago, USA; 3 Querrey Simpson Institute for Bioelectronics, Northwestern University, Evanston, USA; 4 Department of Dermatology, Northwestern University Feinberg School of Medicine, Chicago, USA; 5 Northwestern University, Chicago, IL USA; 6 Department of Surgery, Northwestern University, Chicago, IL USA

**Keywords:** Diagnostic markers, Respiratory signs and symptoms, Epidemiology, Viral infection

## Abstract

There have been over 769 million cases of COVID-19, and up to 50% of infected individuals are asymptomatic. The purpose of this study aimed to assess the use of a clinical-grade physiological wearable monitoring system, ANNE One, to develop an artificial intelligence algorithm for (1) cough detection and (2) early detection of COVID-19, through the retrospective analysis of prospectively collected physiological data from longitudinal wear of ANNE sensors in a multicenter single arm study of subjects at high risk for COVID-19 due to occupational or home exposures. The study employed a two-fold approach: cough detection algorithm development and COVID-19 detection algorithm development. For cough detection, healthy individuals wore an ANNE One chest sensor during scripted activity. The final performance of the algorithm achieved an F-1 score of 83.3% in twenty-seven healthy subjects during biomarker validation. In the COVID-19 detection algorithm, individuals at high-risk for developing COVID-19 because of recent exposures received ANNE One sensors and completed daily symptom surveys. An algorithm analyzing vital parameters (heart rate, respiratory rate, cough count, etc.) for early COVID-19 detection was developed. The COVID-19 detection algorithm exhibited a sensitivity of 0.47 and specificity of 0.72 for detecting COVID-19 in 325 individuals with recent exposures. Participants demonstrated high adherence (≥ 4 days of wear per week). ANNE One shows promise for detection of COVID-19. Inclusion of respiratory biomarkers (e.g., cough count) enhanced the algorithm's predictive ability. These findings highlight the potential value of wearable devices in early disease detection and monitoring.

## Introduction

More than 769 million cases and 6.9 million deaths have been attributed to the severe acute respiratory syndrome coronavirus 2 (SARS-CoV-2) or COVID-19, since the onset of the pandemic^1^. As 50% of infected individuals are asymptomatic and viral shedding occurs 5–6 days before symptom onset, pre-symptomatic transmissibility is one of the greatest causes of widespread disease^2–4^. Laboratory confirmation of diagnosis by reverse transcription-polymerase chain reaction (RT-PCR), though considered the gold standard, is most often performed after symptom onset and is not scalable or practical for daily testing. Results can require up to 72 h of turnaround time and are not reliable at detecting early or asymptomatic disease^5^.

Wearable devices—non-invasive technologies mounted on the human body that continuously capture physiological data—are of growing interest as a resource to understand individual well-being and infectious disease spread^6–10^. These devices gained greater traction during the pandemic^11^. Fitness trackers and smartwatches, worn by 20% of Americans^12^, emerged as naturally scalable methods to remotely monitor patients in real-time. Further inquiry demonstrated subtle changes in biomarkers may signal impending or worsening infection, informing targeted testing, contact tracing, isolation, or escalation of care without risking exposure to the overburdened healthcare system.

A low-profile system aiding in pre-symptomatic detection could mitigate the spread of new infections^8,13^. Previous studies leveraged different wearable platforms to detect pre-symptomatic or asymptomatic carriers of disease^10,14–16^. These studies suffered from few positive cases, self-reporting alone, and retrospective design. Furthermore, many wearable systems used finger or wrist-mounted fitness or wellness devices and were not designed or optimized to capture critical biomarkers relevant to a respiratory virus, including pulse oxygenation, cough count, respiratory rate (RR), and effort, potentially limiting confidence in predictive algorithms.

Earlier identification of disease by comprehensive physiological biomarkers, including those specific to respiratory illness, prior to symptom onset may help contain asymptomatic spread of disease and expedite initiation of medications. Moreover, systems providing real-time physiological data in diagnosed individuals can identify those needing higher acuity care. A South African study of high-risk COVID-19 patients using a pulse oximeter after confirmed infection had lower mortality rates compared to other high-risk patients^17^.

The purpose of this study was twofold, (1) develop an artificial intelligence (AI) algorithm for cough detection in a clinical-grade, FDA-cleared physiological wearable monitoring system (ANNE One, Sibel Health) and (2) develop an AI algorithm for detection of PCR confirmed COVID-19 infection.

## Methods

### Novel respiratory biomarker validation^18^

The ANNE system has multiple FDA clearances (K211305, K220095, and K223711) for monitoring the following parameters: heart rate (HR), RR, pulse oxygenation, skin temperature, body temperature, apnea–hypopnea index, total sleep time, core body position, and snoring in adults^19–22^. Previous studies describe the system’s validation^23–26^.

Cough is a key symptom of COVID-19. We undertook development of an AI algorithm for automated cough detection and count. Healthy subjects at least 18 years old were recruited to wear the ANNE One sensor at the suprasternal notch on the chest and complete a series of activities simulating physiological and environmental signals.

Only features from the chest sensor were used during participants’ performance of scripted activities, including coughing, talking, and resting. Manually labeled coughs were considered ground truth and used to assess the accuracy of the algorithm. Analysis was performed of events to refine the algorithmic discrimination of cough using accelerometry from the chest sensor.

The algorithm used a single channel 400–1600 Hz accelerometer with the axis normal to the attachment surface. The high frequency channel captured the acousto-mechanic signal of vocal and respiratory activity. The signal was filtered and converted into audio files. Researchers manually listened to waveform audio files obtained from the accelerometer signal to identify and label coughing events. Inclusion of non-coughing, potential confounding activities ensured that the level of uncertainty driven by these activities fell within an acceptable range. The labeled data were randomly split into training and testing sets in 3:1 ratio by subject. We trained a convolutional recurrent neural network. We applied a short time Fourier transform on the 1666 Hz accelerometer z-axis data and took the log magnitude. The length of the short-time Fourier transform (STFT) was 128 and the overlap was 64. The model computed a cough probability every 0.192 s. For training, we labeled a time step as cough if the previous 1 s data was annotated as cough. The cough index was calculated as the average cough probability over a second. Since most of the STFT features were above the frequency range of motion, our model was not sensitive to motion artifacts.

### Home-based COVID-19 detection program

We performed a multicenter single arm prospective study to describe the predictive capacity of an AI-enabled algorithm using ANNE One system outputs for early detection of PCR-confirmed COVID-19 infection among a cohort of subjects at high infection risk. Participants were eligible if they were 18 years or older and if they had an occupational or home exposure to an individual with a new diagnosis of COVID-19 infection within the past 7 days. Enrolled patients had to be able and willing to provide written consent and comply with the study’s procedures. Patients were excluded if they were pregnant, nursing, or planning pregnancy. Individuals with prior known COVID-19 infection and complete symptomatic resolution or with known active COVID-19 infection were excluded.

To recruit individuals with exposures, patients who tested positive for COVID-19 at Northwestern Memorial Hospital or Northwestern University Health Service COVID-19 testing centers were provided study and recruitment information to share with interested household contacts or caregivers. Additionally, healthcare providers caring for COVID-19 positive patients were also informed about the study.

At the time of enrollment, participants provided demographic information and baseline medical history including smoking history, previous history of COVID-19 infection, number, date, and type of COVID-19 vaccination(s), and co-morbidities. Subjects underwent baseline PCR-testing for COVID-19 with a nasopharyngeal swab performed by trained study coordinators. Participants were offered an optional enzyme immunoassay for SARS-CoV-2 antibodies from red blood spot samples by either fingerstick or peripheral blood draw^27^.

Study coordinators completed in-person or virtual training on application and wear of the wireless sensors. Participants were instructed to wear the chest sensor as consistently as possible, including during regular daytime activities and sleeping. The limb unit was worn while sleeping and intermittently throughout the day as much as was convenient to subjects. Subjects were provided a sensor charger and tablet preloaded with the companion application. Subjects removed the sensors once daily to recharge the device. Data upload occurs concurrently with device charging.

Subjects completed electronic surveys of COVID-19 symptoms, inquiries about interval COVID-19 diagnosis and COVID-19 vaccination, and the Wisconsin Upper Respiratory Symptom Survey daily. Participants without data uploads or surveys for 2 or more consecutive days were contacted by a study coordinator.

Full study participation concluded after monitoring for 4 weeks. Participants underwent repeat PCR COVID-19 testing via nasopharyngeal swab, an optional blood antibody test for COVID-19, and completed a survey on their experiences with the sensors. Study subjects concluding participation early underwent repeat nasopharyngeal COVID-19 PCR testing if participation exceeded 1 week. The electronic medical record (EMR) was reviewed for interval infection and vaccination receipt.

All specimens collected for COVID-19 testing were processed as received and were analyzed per the hospital’s universal policy for all clinically collected COVID-19 swabs. The study was approved by the Institutional Review Board at Northwestern University (Protocol #STU00213040), and all methods were performed in accordance with relevant guidelines and regulations. All participants provided written informed consent. The datasets used and/or analyzed during the current study available from the corresponding author on reasonable request.

### Statistical analysis

The primary endpoint of the COVID-19 detection program was to develop AI-algorithms based on labeled data with positive and negative COVID-19 infection. We conducted a retrospective analysis of all data endpoint outputs from the sensors and survey results for all COVID-19 positive individuals prior to molecular diagnostic confirmation and age matched negative controls from the same cohort who tested COVID-19 negative. Subsequent primary outcomes included determination of the sensitivity, specificity, and accuracy of asymptomatic, pre-symptomatic, or early symptomatic COVID-19 infection detection by the ANNE One system and PCR testing. Sample size was determined from a power analysis with each subject generating 336 h of data with 48 positive cases expected. Assuming a secondary attack rate of 15%, we required at least 48 positive cases to train our machine learning algorithms reaching 322 subjects. A sample size of 322 would achieve an 80% power for a Pearson correlation of 0.8 assuming significance of 0.05.

## Home-based COVID-19 detection program algorithm development

### Data collection

For each raw data collection session, skin temperature, HR, heart rate variability (HRV), RR, pulse rate, perfusion index, physical activity, pulse oximetry, speech, and cough activity were generated at one-second intervals. Data with low signal quality or data collected during times when the sensors were unattached were eliminated from the model. To minimize the effect of missing data due to malposition, lost leads, or poor signal quality, we used a grid representation of the time series as the input for the model. Two hour long vital snapshots were taken with 30-min intervals. Each interval had a 90-min overlap between adjacent data points. These snapshots were then labeled based on the subject’s PCR and antibody testing results (as ground truth) from the beginning and end of the study and the time of data collection with respect to these results. Each vital parameter was then mapped into a 2D grid matrix with predefined dimension. Any data with missing testing results or snapshots with more than 50% of data missing were discarded from classification.

### Variable labeling

We created an expanded label set for all observed possible daily outcomes for subjects to better reflect complex health status changes throughout the study. Subjects were labeled based on changes in COVID-19 PCR testing, antibody status, symptomatic report, and interval receipt of vaccinations from baseline compared to study exit. Subjects without symptoms, negative PCR testing, and no change in baseline antibody status were designated *NN*. Subjects with positive baseline or exit antibody testing, but otherwise negative PCR testing and no symptoms were also defined as *NN.* Infection cases were designated as *P* if subjects were symptomatic and had positive PCR testing upon study enrollment. Subjects with self-reported mild to severe symptoms and a documented change in antibody status without interval vaccination were also designated as *P*. Subjects with self-reported mild to severe symptoms but without confirmatory testing were labeled *NP*. Those with antibody change but without record of vaccination nor without noticeable symptoms were labeled as *U*. Afterwards those with negative PCR results at the end of the study are labeled as *N*. Those with insufficient survey and lab testing results to perform imputations were labeled as *X*. The *NN* class was used as the negative class, and the *P* class was used as the positive class during training. The reported accuracy was using the average probability in the first 5 days to predict the PCR test result on the first day.

**Figure 1:**
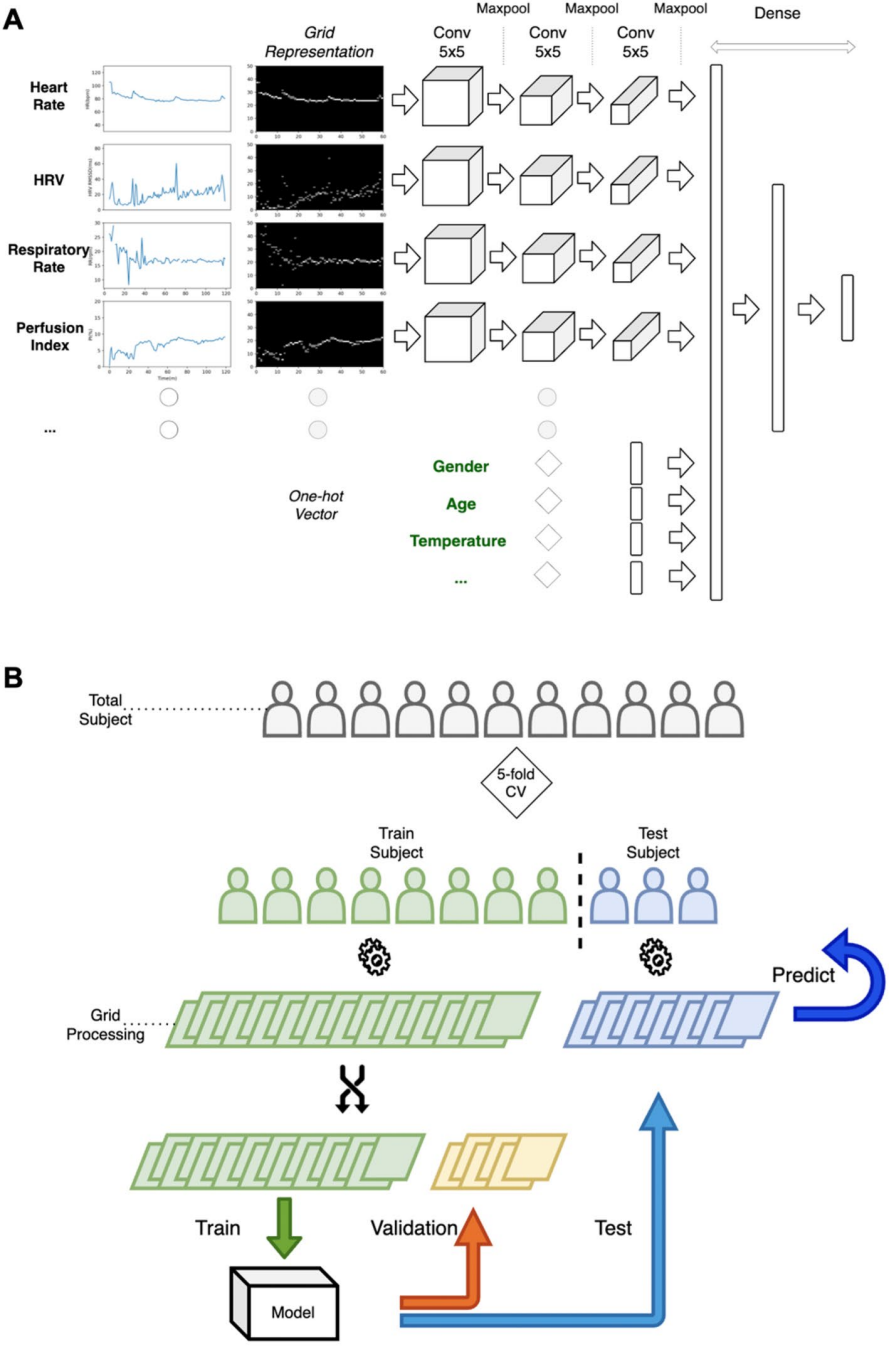
This figure demonstrates generation, validation, and testing of the model. In panel (**A**) the grid representation of each vital is fed into individual convolution modules. Additional information such as the subject's gender, age, and vitals without significant time varying components were fed in as one-hot vectors. Temperature values were rounded to the nearest integer. Chest temperature greater than or equal to 30 Celsius and smaller than 40 Celsius, and limb temperature greater than or equal to 28 Celsius and smaller than 38 Celsius are one-hot coded (values outside of the range would be set to zero, otherwise the value is set to one). In panel (**B**) we demonstrate that subjects were split into train and test groups with five-fold cross validation. Datasets for subjects in the training group were further split into training and validation sets for hyperparameter tuning and overfitting detection. Training datasets were used to create the convolutional neural network model and to evaluate which features generate the highest predictive value.

Subjects were split into train and test groups with five-fold cross validation. Datasets for subjects in the training group were further split into training and validation sets for hyperparameter tuning and overfitting detection. Training datasets were used to create the convolutional neural network model and to evaluate which features generate the highest predictive value (Fig. 1).

## Results

### Respiratory biomarker validation

**Figure 2:**
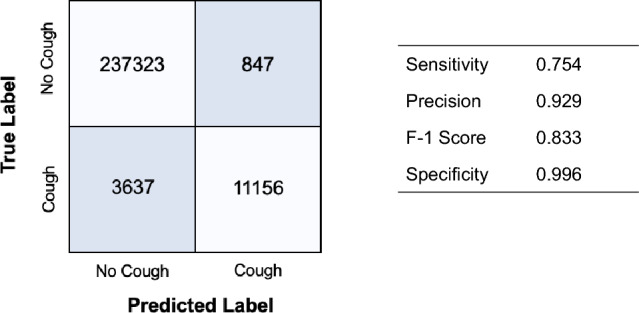
The tables present performance of the cough detection algorithm via analysis of the sensor’s accelerometer signal. Final performance of the test data demonstrated a F-1 Score of 83.3%.

A study was conducted in 27 healthy subjects to evaluate the functionality of the ANNE Chest sensor for cough detection. An automated algorithm quantified cough through analysis of the chest accelerometer signal demonstrating final performance on the test data of 83.3% for the F1 score (Fig. 2).

### Home-based COVID-19 detection program

Between November 5, 2020, and March 31, 2021, a total of 325 patients were enrolled generating 69,170 h of data collected from the sensors. The study population consisted of 62.2% (n = 202) women, and 62% self-identifying as White, 14% as Asian, and 12% as Black. The mean age of participants was 29.4 years (SD 12.2) (Table 1). Overall, 92% of participants wore the sensors for at least 4 days per week and 99% wore the sensors for at least 3 days per week.

**Table 1:** This table displays basic demographic information for enrolled subjects.

Factor	Value
N	325
Age, mean (SD)	29.4 (12.2)
Age distribution	
< 25 years	127 (41.1%)
25–35 years	111 (35.9%)
35–45 years	12 (3.9%)
45–55 years	22 (7.1%)
> 55 years	37 (12.0%)
Sex	
Female	202 (62.2%)
Male	122 (37.5%)
Prefer not to identify	1 (0.3%)
Race	
White	202 (62.2%)
Asian	44 (13.5%)
Black	40 (12.3%)
American Indian/Alaskan Native	2 (0.6%)
More than one race	19 (6.0%)
Unknown	2 (0.6%)
Choose not to identify	10 (3.1%)
Ethnicity	
Hispanic or Latino	33 (10.2%)
Not Hispanic or Latino	277 (85.2%)
Unknown	15 (4.6%)

**Figure 3:**
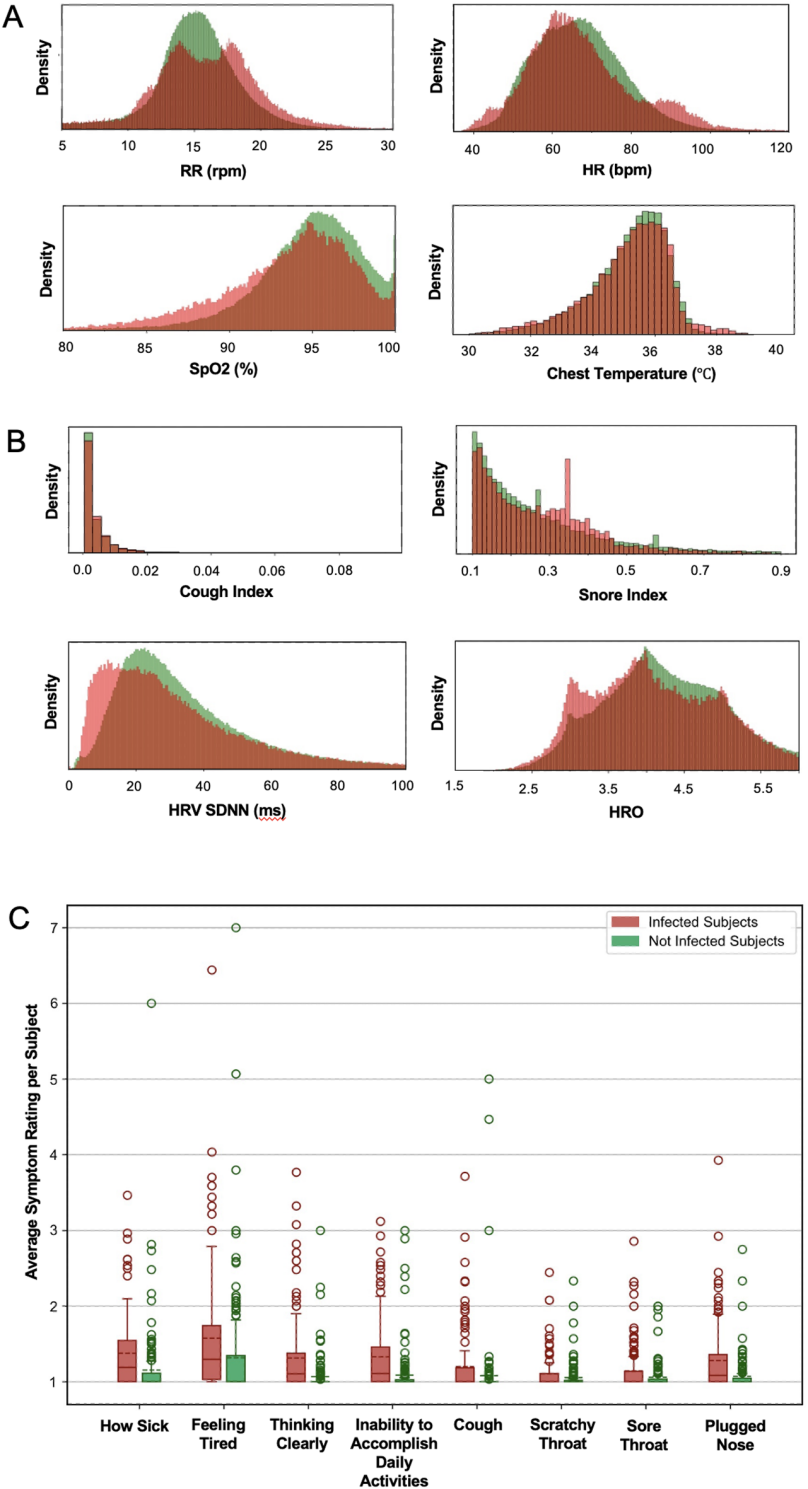

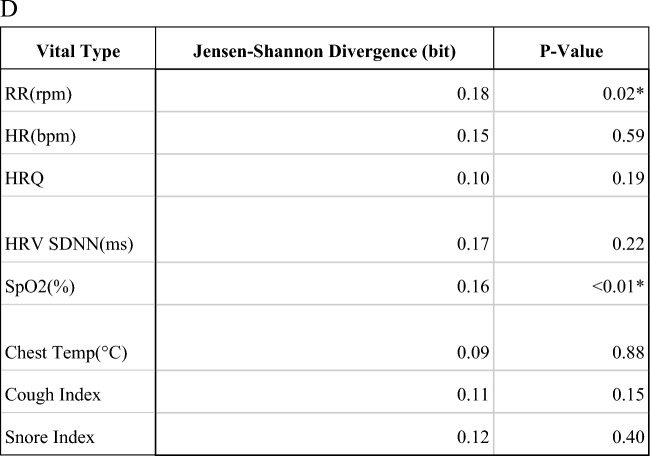
This figure demonstrates the variable vital sign distribution (**A**), (**B**), and self-reported symptoms (**C**) distribution among those subjects who were infected (red) and non-infected (green). The Jensen-Shannon Divergence and P-value of each vital type is presented (**D**). The *P*-values of the vitals except cough index and snore index are calculated using a clustered *T*-test. The *P*-values of cough index and snore index are calculated using a clustered Wilcoxon rank sum test. The figures demonstrate a measurable difference in objective and subjective measures obtained by the ANNE system.

At baseline, of the 325 patients enrolled, 268 underwent PCR testing, demonstrating a positivity rate of 23.1% (n = 75 patients). Of those not formally tested, an additional 20 patients self-reported positive COVID-19 testing results, for an overall baseline positive rate of 29.2%. Furthermore, 33.5% (n = 109 of 316 tested) of participants were antibody positive. At the conclusion of the study 4.3% (n = 14) had positive PCR testing, and 49.5% (n = 161 of 273 tested) had a positive antibody status. A total of 22.8% (n = 74) of patients seroconverted by the conclusion of the study. Overall, 16 individuals (n = 4.9%) received interval COVID-19 vaccinations. An additional 15 participants (n = 4.6%) were excluded due to insufficient or missing data. Variable distributions of vital signs by disease status were compared (Fig. 3).

**Figure 4:**
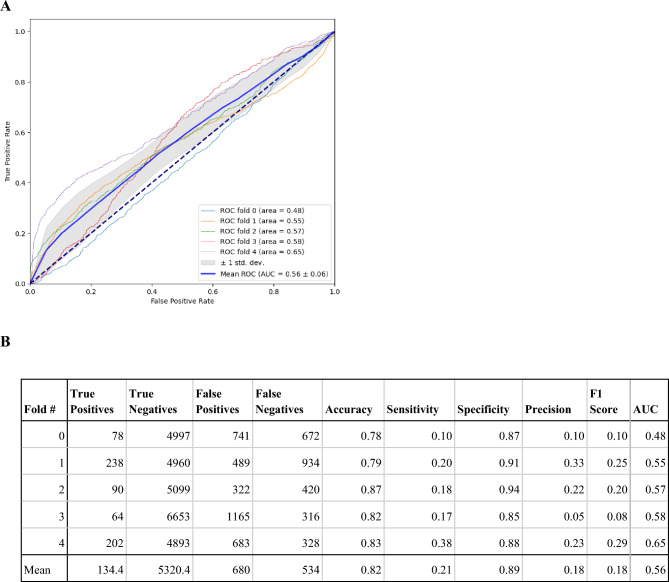
(**A**) The performance of the ANNE system for prediction of COVID-19 in 2-h data windows is illustrated in the receiver operating (ROC) curves shown in panel (**A**) with the corresponding sensitivity, specificity, precision accuracy, AUC, and F1 score for each fold in panel (**B**). We conducted a subject-wise fivefold cross validation using class P as the positive class, and class NN as the negative class. The model is trained to predict the class label for each 2-h window.

**Figure 5:**
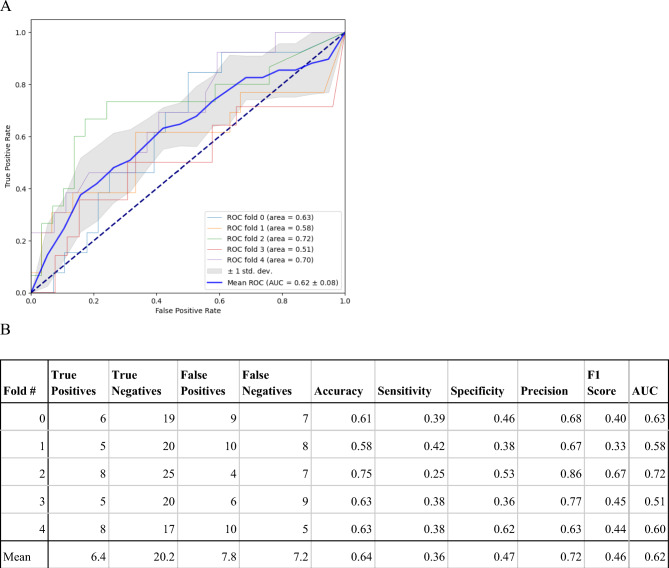
(**A**) The performance of the ANNE system for prediction of COVID-19 compared to the gold standard PCR test is illustrated in the receiver operating (ROC) curves shown in panel (**A**) with the corresponding sensitivity, specificity, accuracy, precision, AUC, and F1 score for each fold in panel (**B**). Using the trained model (performance shown in Fig. 4), we took the average class P probability of all 2-h data windows during the first 5 days of the study for each subject in the test set. The average probability is used to predict the PCR test result on the first day.

We conducted a subject-wise fivefold cross validation using class P as the positive class, and class NN as the negative class. The mean area under the curve (AUC) for the receiver operating (ROC) is 0.56 ± 0.06 (Fig. 4). Using the trained model, we took the average class P probability of all 2-h data windows during the first 5 days of the study for each subject in the test set. The average probability is used to predict the PCR test result on the first day. The predictive capabilities in the ANNE One system compared to the COVID-19 PCR test is illustrated in the ROC. The mean AUC for the ROC is 0.62 ± 0.08 (Fig. 5). The accompanying table includes sensitivity, specificity, accuracy, AUC, and F1 score for each fold. The developed algorithm had a sensitivity of 0.47 and specificity of 0.72 for detection of asymptomatic, pre-symptomatic, or early symptomatic COVID-19 infection.

To assess the difference in performance on asymptomatic, pre-symptomatic and symptomatic data windows, we created a new set of labels for the test data based on the PCR test results at the enrollment and the end of the study. Those with positive PCR testing at the enrollment and the end of the study are labeled positive. Those with negative PCR testing at the enrollment and the end of the study are labeled negative. For the rest of the patients, they are labeled according to the PCR result for the 14 days following the enrollment and the 7 days prior to the end. The model performance is summarized in Table 2.

**Table 2:** Comparison of model performance on asymptomatic, pre-symptomatic, and symptomatic data windows, where TP (true positive), FP (false positive), TN (true negative), FN (false negative).

Fold #	Asymptomatic					Pre-symptomatic					Symptomatic				
	TP	TN	FP	FN	F1	TP	TN	FP	FN	F1	TP	TN	FP	FN	F1
0	0	1168	167	0	0	1	690	167	6	0.0114	160	2108	285	955	0.205
1	25	1220	90	167	0.163	0	616	72	0	0.0000	267	2950	325	957	0.294
2	13	933	116	51	0.135	20	666	78	162	0.1429	181	3042	109	1092	0.232
3	27	972	112	144	0.174	0	351	19	0	0.0000	202	3962	502	1026	0.209
4	1	972	91	11	0.019	0	745	42	0	0.0000	296	2260	258	1117	0.301
Mean	13.2	1053	115.2	74.6	0.098	4.2	613.6	75.6	33.6	0.0309	221.2	2864.4	295.8	1029.4	0.248

Over the course of the study, two syncopal events were recorded when subjects self-administered a finger stick for a COVID-19 antibody test. No other serious adverse events were noted outside of temporary skin irritation in 8 subjects (2%).

## Discussion

This study developed an algorithm to detect cough and a second algorithm to detect infection with COVID-19 among a cohort of high-risk subjects with recent environmental or occupational exposures.

The cough detection algorithm described herein is based on a reusable flexible, accelerometer-based sensor, worn at the suprasternal notch, achieved an accuracy of 98% for cough detection, with 75% sensitivity and greater than 99% specificity in twenty-seven healthy subjects during biomarker validation. This performance is comparable with prior reports but achieves detection via a low-profile chest sensor^28,29^. Automation of objective cough detection by wearable devices has potential benefits for patients for patients with chronic respiratory effects and acute infections. Sensors measuring general physiological parameters in addition to more novel respiratory biomarkers, such as cough, may have improved detection of infectious processes or disease identification.

The developed COVID-19 detection algorithm incorporating heart rate, body temperature, respiratory biomarkers, and daily patient-reported symptoms, demonstrated a sensitivity of 0.47 and specificity of 0.72 for detection of COVID-19 infection. We demonstrated high acceptability of the ANNE One sensors given high adherence and daily wear, despite previous studies suggesting a preference for wrist-mounted wearables. The chest sensor provided respiratory biomarkers unobtainable by finger or wrist mounted devices potentially improving algorithm accuracy.

Wearable devices as tools for remote monitoring have been previously reported. A South African study of high-risk, infected patients monitoring pulse oxygenation at home compared to those who did not demonstrated a mortality benefit, attributed to earlier hospital presentation^17^. Wearables may also mitigate disease spread via detection of pre-symptomatic or asymptomatic disease^30^. Early studies used single biomarkers (e.g. Temperature or RR) to identify known, early symptomatic cases of COVID-19 and relied on retrospective self-reported timing of symptoms and testing^30,31^. A retrospective study with Fitbit found abnormal resting HR as early as 7 days prior to symptom onset in 23/25 self-reported positive cases^16^. Mishra et al. similarly reported an HR, step count, and sleep data from Fitbit smartwatches demonstrated f63% of COVID-19 cases had aberrant signals prior to symptom onset (n = 32)^14^. In the TemPredict study, physiological data from the Oura Ring, developed an algorithm identifying COVID-19 2.8 days before participants pursued testing (sensitivity of 82% and specificity of 63%)^32^. Dried blood spots were used for antibody testing, and derivation of RR via photoplethysmography may compromise accuracy compared to chest wall movement, particularly at higher respiratory rates. Algorithms did not incorporate pulse oxygenation or cough count^33^. In a cohort of 3318 individuals (of whom 84 contracted COVID-19), HR and step count abstracted from any smartwatch and combined with patient-reported symptoms successfully generated an alert for 80% of COVID-19 cases at a median of 3 days prior to symptom onset^34^.

Furthermore, a secondary analysis from the Digital Engagement and Tracking for Early Control and Treatment (DETECT) study, a large, prospective app-based, device agnostic cohort of individuals who electively shared data and self-reported symptoms, testing, and vaccination status, demonstrated biomarkers from wearable devices showed deviations in HR, sleep and activity, predicting reactogenicity and immune response following COVID-19 vaccination^35^. A secondary analysis of the TemPredict Study similarly found deviations in resting HR, HRV, and temperature measured by the Oura ring predicting post-vaccination antibody levels^36^.

This is one of the first studies leveraging FDA-cleared biomarkers from a wearable to provide early detection of COVID-19 using molecularly confirmed positive RT-PCR COVID-19 testing^14,15,30,31,37^. The TemPredict study performed SARS CoV-2 antibody testing with dried blood spots which though offered confirmatory laboratory diagnosis, is not the gold standard and was only available in a small proportion of patients (3664 participants of 63,000 enrolled)^32^. In addition to molecularly confirmed disease, the presented study is one of the first to incorporate respiratory biomarkers into a predictive algorithm with all core vitals FDA-cleared. The ANNE One System collected more biomarkers, including novel respiratory vital signs not captured by wristwatches or rings (e.g. cough count) with value in determining infectiousness based on cough frequency^38^. A chest-mounted system, less sensitive to ambient temperature changes, may more accurately measure body temperature. Previous studies show some patients have an unstable baseline, and higher-resolution, information dense models may improve accuracy and specificity of algorithm performance^34^.

There are important limitations to this study. Though the study utilized laboratory testing to identify positive cases, the specific variant of COVID-19 was not determined. Patients were recruited primarily during the Delta wave in 2020. Subsequent variants, such as Omicron, may have variable biological presentations with mild disease and less robust biological signals, affecting sensitivity and specificity of the algorithm^39^. Additionally, it is possible some positive cases of COVID were misclassified as negatives given missing laboratory data. Efforts were made to mitigate the risk of misclassification through serological testing and interrogation of the EMR. Collection of interval vaccination was performed to prevent misclassification. The study only documented testing for COVID-19 and it will be important to build libraries of patients with other diseases. The ability of the algorithm to distinguish COVID-19 from other infectious or respiratory diseases (e.g. Asthma) was outside the study’s scope.

As we progress to new stages of the COVID-19 pandemic, devices serving multiple roles from asymptomatic disease detection, monitoring well-being in the setting of known infections, and measures associated with immunization response will become only more relevant. Low-profile devices measuring respiratory biomarkers and incorporating patient symptoms may be best suited to build scalable, population-level pre-symptomatic detection programs and assess for deterioration of infected individuals.

## Data Availability

The data for the development of a cough detection algorithm and COVID-19 study are available upon request to Shuai Xu MD at stevexu@northwestern.edu.
